# Intracranial Tumours

**Published:** 1888-12

**Authors:** 


*Intracranial Tumours.* By BYROM BRAMWELL, M.D. Pp. 170. Edinburgh: Y. J. PENTLAND. 1888.

Dr. Bramwell's clinical memoirs are always well worthy of perusal. This one, on intracranial tumours, although its foundation is almost purely clinical, and based on the author's keen and exhaustive observation, is also, as embodying much generalised and philosophic doctrine, a somewhat ambitious treatise on the whole subject of cerebral tumours. The work is in every sense commendable. It includes the latest researches and theories, is well balanced, clearly written, and abundantly and most admirably illustrated. The tendency of the day is seen in the surgical bent of the book, which has its practical outcome in an excellent chapter on Surgical Treatment of Intracranial Tumours, written by Mr. A. W. Hare.

